# The Challenges of Ethical Review in Clinical Research of Traditional Chinese Medicine

**DOI:** 10.1155/2021/6754985

**Published:** 2021-11-12

**Authors:** Jie Zhang, Zong-Ming Zhang

**Affiliations:** ^1^Institute of Literature in Chinese Medicine, Nanjing University of Chinese Medicine, Nanjing 210023, China; ^2^Nantong University Xinglin College, Nantong 226236, China; ^3^Research Center of Chinese Medicine Culture, Nanjing University of Chinese Medicine, Nanjing 210023, China

## Abstract

The ethical review of TCM (traditional Chinese medicine) clinical research in China is highly consistent with that of Western medicine, but it lacks the characteristics and culture of TCM. Compared with modern medicine, TCM has its own characteristics, such as the theory of Yin-Yang and the five elements, the treatment of syndrome differentiation, and the compatibility of prescriptions. These characteristics determine the ethical particularity of TCM clinical research. This article discusses the challenges in the ethical review of TCM clinical research, such as scientific design, efficacy evaluation, risk assessment, informed consent, and placebo use. We propose opportunities and difficulties in the ethical review of TCM clinical research and provide some relevant suggestions.

## 1. Introduction

The development of medical ethics can be traced back to the Nuremberg Trial after World War II. The promulgation of the Nuremberg Code put forth the first international file of ethical principles for medical trials involving human beings. In 1964, the Joint Congress of the World Medical Associations issued the Declaration of Helsinki, the second international file for human trials, and a series of ethical principles were established, such as the legitimacy of the research purpose, scientificity of the research methods, protection of participants' interests, and fully informed consent. To date, the Declaration of Helsinki has been revised by several sessions of the Joint Congress of the World Medical Associations. These ethical principles have been stipulated by an increasing number of countries as the ethical norms of human trials that protect the rights of participants and make medical research more standardized and reasonable. In 2007, the Chinese government issued the “Methods for the Ethical Review of Biomedical Research Involving Humans (Trial),” which provides a standard ethical review for biomedical research involving humans in China. In 2016, formal “Methods for the Ethical Review of Biomedical Research Involving Humans” was promulgated [1]. Traditional Chinese medicine (TCM) is an important part of mainstream medicine in China. It has also attracted increasing international interest for its global use, potential impact on healthcare, and opportunities for new drug development. Compared with Western medicine, TCM has its own characteristics, such as the theory of Yin-Yang and five elements, treatment of syndrome differentiation, and compatibility of prescriptions. These characteristics determine the ethical particularity in TCM clinical research. In 2008, the State Administration of Traditional Chinese Medicine initiated the standard of ethical review of TCM clinical research by successively issuing “Practices of Ethical Review of TCM Clinical Research” [2]. In 2011, China set up the Institutional Review Board for traditional Chinese medicine (IRB) to conduct the review. The ethical principles in “Practices of Ethical Review of TCM Clinical Research” are consistent with the ethical review of Western medicine in general, but they lack the characteristics of TCM. General ethical principles are the foundation of TCM ethical review, and special attention to the scientificity of the research design, efficacy evaluation, risk assessment, informed consent, and placebo use should also be paid due to the characteristics of TCM.

## 2. Scientific Validity

Some ethics committee members believe that the duty of the ethical review is only to review ethically and not scientifically. In fact, any clinical research that has scientific problems must be unethical. Scientificity is the precondition for clinical research to be ethical. Scientific validity also determines the value of the research for society. The challenges in scientific validity include the particularity of TCM theory, quality control of Chinese herbal medicine, and methods for efficacy evaluation.

### 2.1. The Particularity of TCM Theory and Research Design

In evidence-based medicine (EBM), randomized controlled trials (RCTs) are often considered the most scientific and rigorous method of hypothesis testing [3]. However, a randomized controlled design may not be suitable in research on TCM for individualized treatment due to the particularity of TCM theory. The theory of TCM originates from the ancient Chinese philosophy of Qi, Yin-Yang, and the five elements. Based on the four diagnostic methods of TCM and syndrome differentiation, the patients were treated with Chinese medicine, acupuncture, massage, cupping, and Qigong. The “syndrome” in “syndrome differentiation” refers to the conditions of pulse, tongue, body and spirit, complexion, and other related symptoms presented by the patients, which reflect the cause, nature, and stage of the disease. For example, “The Sixth Edition of COVID-19 Diagnosis and Treatment Protocol” released by the National Health Commission in China divided the treatment period into the light type, common type, severe type, critical type, and convalescence period [4]. According to syndrome differentiation by TCM, COVID-19 is also divided into four stages: early stage, middle stage, severe stage, and recovery stage. In the early stage, it appears to be an exterior syndrome of cold and dampness affecting the lung and spleen or dampness stagnating heat. The treatment is ventilating lung qi, clearing damp turbidity, and dredging collaterals. In the middle stage, pathogenic factors invade the interior of the body and can be divided into two types: syndrome of heat toxicity blocking the lung (more heat with less dampness) and syndrome of accumulating dampness-toxicity retention in the lung (more dampness with less heat). The treatment for syndromes of heat toxicity blocking the lung is clearing heat, removing toxicity, diffusing the lung, and purging viscera. For syndromes of accumulating dampness-toxicity retention in the lung, the emphasis is on dissipating dampness. In the severe stage, heat enters nutrients and the blood, and inner blocking causes collapse. The treatment is clearing heat and relieving turbidity, nourishing yin for lowering fire, and warming yang for relieving desertion. In the recovery stage, the major syndromes are spleen-lung qi deficiency and qi-yin deficiency. The treatment invigorates the lung and spleen, relieves turbidity, and dredges collaterals. Therefore, the performance and treatment protocols in various stages of the same disease are different. However, the diagnosis of “syndrome” is usually subjective, ambiguous, and unrepeatable, and it is difficult to find quantitative indicators even if part of the scientific basis of “syndrome” in TCM has been revealed by related studies with the development and application of genomics and metabonomics in recent years [5–8]. Meanwhile, it is challenging to format an acknowledged and standard model in syndrome differentiation, and the current methods for diagnosis are not perfect either.

The diagnosis and treatment principle of TCM is “syndrome differentiation”. As a result, problems associated with how to design a TCM clinical trial arise, including whether the inclusion criteria are based on disease classification or syndrome classification. In general, clinical trials of chemical drugs are based on the classification of diseases in modern medical research, whereas TCM studies tend to choose syndromes as the first choice. The composition of newly developed prescriptions and the determination of indications are mainly based on the elaboration of drugs in, for example, the “Pharmacopoeia of the People's Republic of China,” authoritative “Guiding Principles for Clinical Research of New Chinese Medicine” [9], “Criteria for the Diagnosis and Curative Effect of TCM Diseases” [10], and unified textbook “Internal Medicine of Traditional Chinese Medicine” [11–13]. The accuracy of the inclusion of syndromes and indications directly affects the outcome of the study. However, these authoritative studies cannot distinguish the syndrome comprehensively, objectively, and accurately. There is still widespread confusion regarding the syndrome name, attribute, quantity, and symptoms in syndrome differentiation for common diseases, such as coronary heart disease, hypertension, and stroke [14, 15]. Therefore, it is difficult to reach an international consensus on the inclusion and exclusion criteria of RCTs in TCM clinical studies. When TCM studies are in accordance with Western disease standards, if the test drug does not match the participant's syndrome in the research process, the increasing risks lead to potential safety problems.

Because of the differentiation and individualization of TCM theory and clinical practice, it is difficult to design a generally recognized model for TCM research. If the ethics committee is not familiar with the theory of TCM, the relevant ethical review will be difficult. In turn, the difficulty of ethical review also brings challenges for researchers, so they need to submit innovative designs to adapt these features of TCM. Some researchers have used the method of combining modern medical inclusion criteria with TCM syndrome differentiation to design the trial. For example, in the design of a multicentre, randomized, double-blind, placebo-parallel controlled trial for the efficacy and safety of shenfuqiangxin pills in the treatment of chronic heart failure, patients were included in the trial with the heart-kidney yang deficiency syndrome, such as heart palpitations, shortness of breath, and fear of cold [16]. The design reflects the characteristics of TCM and the efficacy evaluation of Western medicine. Other investigators have used a multiarm design in TCM research, such as comparing the clinical efficacy using a personalized approach, a fixed approach, and a placebo control in one trial [17, 18]. Similar approaches would be more ideal in science research for increasing the validity of the results. Researchers of TCM may not be used to designing TCM research under the paradigm of Western research methods. It requires that researchers and ethics committees learn from each other and engage in ongoing communication, which is an important strategy for fulfilling the obligations of both parties successfully; otherwise, they may find it difficult to seek ethical recognition on both sides.

Because of the individualization and nonrepeatability of TCM, real-world studies (RWSs) have come into view in recent years. An RWS is a new method for TCM clinical studies that focuses on individualized diagnosis and treatment effects in the real world. It has broader inclusion and exclusion criteria that cover a wider range of people, and the results are closer to real clinical practices [19]. An RWS can address the limitations of poor conclusion extrapolation and a lack of medical ethics in some cases of RCTs. It can also take advantage of TCM syndrome differentiation and integrated treatment methods in exploring the relationship between clinical treatment and TCM theory and draw convincing and significant conclusions as RCTs do. The core ethical issues faced by RWS are data-related ethics, including privacy protection and data security. These problems can be better solved through relevant legislation and policy formulation.

### 2.2. The Particularity of Chinese Herbal Medicine and Quality Control

The formation and development of TCM are closely related to the long history of traditional Chinese culture. The theory of “principle, method, prescription, and medicine” is permeated with profound ancient Chinese philosophy. For Chinese herbal medicine (CHM), the theory of “nature, taste, and meridian tropism” is deduced from ancient philosophy based on a large number of clinical observations and guides clinical practices in return. The principles of Chinese medicine formulation have been stipulated in “Sheng Nong's Herbal Classic” and “Huangdi's Internal Classic” as “monarch, minister, assistant, and guide.” TCM regards each medicinal material as a single and indivisible “individual” and classifies the pharmacological effects as “cold, hot, warm, and cool,” “up and down,” and “sour, sweet, bitter, and salty” according to its “nature” and “taste.” Chinese herbal compound prescription is a “group” composed of several individuals, which can be summed up as the relationship of “monarch, minister, assistant, and guide,” so that each of them performs its own duties and, altogether, they play an inseparable overall role.

Compared with chemical drugs, CHM has unique advantages in some aspects as well as disadvantages and deficiencies in other aspects. The chemical compositions of CHM are very complex, including the effective components and auxiliary components in the cure of diseases and ineffective components. The efficacy of CHM does not come from any single active ingredient but from the combined effect of multiple active ingredients or even synergistic action with “inactive ingredients.” One single ingredient cannot represent one single herb, nor can a single effect represent all its functions. An effective ingredient in treating one disease may be ineffective in treating another. In this sense, a single herb is a small compound. In addition, CHM is derived from nature. Due to the different growth environments, harvest seasons, processing methods, and storage conditions, the same kind of herb varies greatly in chemical composition, quality, and even clinical efficacy. Without quality control, TCM clinical trials may be disqualified due to a lack of scientific validity and risk-benefit analysis. Since the implementation of Good Agricultural Practice for Chinese Crude Drugs (GAPs) in 2002, more than 100 kinds of commonly used CHMs, including *Salvia miltiorrhiza*, *Astragalus membranaceus*, *Panax notoginseng*, *Glycyrrhiza uralensis*, and *Isatis tinctoria*, have been cultivated through standard operating procedures (SOPs) [20]. Research on the chemical and phytochemical analysis of TCM has also reached new heights. More than 6,000 new natural ingredients have been isolated and identified from CHM, including a large number of bioactive compounds that may be used in the development of new drugs. Basic research on comprehensive pharmacodynamic substances under the guidance of TCM theory is the cornerstone of the construction of a TCM quality standard system. Compared with foreign monographs on botanical medicine, the most remarkable and important difference of TCM standards in the Chinese Pharmacopoeia is that the establishment of qualitative and quantitative components of TCM standards cannot be separated from TCM clinical function. On the other hand, botanicals are standardized by the existing chemical research bases. For most CHMs, especially compound preparations, neither one nor several active ingredients can reflect the overall efficacy. CHM with multicomponent and multitarget effects requires an overall quality standard system. At present, the core technology of TCM quality control includes high-performance liquid chromatography (HPLC), fingerprint spectroscopy, and quantitative analysis of multiple components by a single marker (QAMS). In the study of the CHM mechanism, which is a complex system, TCM theory should guide the rule as well as participation in multiple disciplines, such as systems biology. Systems biology, including omics technology, such as genomics, transcriptome, proteomics, metabolomics, microbiome, and network pharmacology, can be applied to CHM composition, pharmacology, toxicology, origin identification, cultivation, and genetic breeding research, so its overall view is consistent with the TCM holism concept in some aspects [21].

The globalization of CHM has also made some progress. The United States Pharmacopoeia (USP), European Pharmacopoeia (EP), and other national authorities have compiled standards for many traditional herbs and botanical drugs, among which USP has adopted more than 15 quality standards, whereas EP has included 75 quality monographs [22]. While referring to the English version of the Chinese Pharmacopoeia, other national pharmacopeia committees have formulated the quality standards according to the situations in their own countries. The World Health Organization (WHO) has also published standards for herb processing in the postharvest stage and set technical requirements for quality control in the production of herbs [22]. These mechanisms could strengthen quality standards for herbs in clinical trials. However, no Chinese medicine prescriptions have obtained marketing approval, especially in the European and American markets, where CHM often appears in the role of healthcare products or food supplements. Therefore, in the process of promoting the internationalization of CHM, Chinese quality standards should guide international standards. Through the construction of a complete quality standard system, CHM can gain international recognition in the European and American markets in the form of medication.

Currently, in the research on new prescriptions, researchers often pay more attention to the chemical compositions but ignore the integrity of the prescription. They focus on the material basis of medicinal effects while ignoring the role of “nature and taste” in prescription compatibility and clinical efficacy. It deviates from TCM theory and the original intention of “formula corresponding to syndrome.” In this manner, the results of the research are separated from the TCM theoretical core and cannot fully explain clinical efficacy. Liu [23] proposed the concept of a quality marker of Chinese materia medica (Q-marker) by integrating multidisciplinary knowledge according to the characteristics of the TCM medical system, such as biological properties, manufacturing process, and compatibility theory, to guide TCM quality control and further strengthen the correlations among TCM efficacy, material basis, and quality control index components. He pointed out that the basic conditions of Q-marker include the following: (1) chemical substances inherent in Chinese materia medica and Chinese medicinal products or formed in the process of medicine preparation, (2) relationship with the functional properties of Chinese materia medica and a clear chemical structure, (3) substances that can be qualitatively identified and quantitatively determined, and (4) according to the principle of Chinese medicine compatibility, monarch medicine is the major target for research, taking into account representative substances of minister and guide medicine. The proposal of quality markers of Chinese materia medica is conducive to the establishment of TCM quality control and quality traceability systems. Zhang et al. [24] also proposed important means and research methods to determine Q-markers in Chinese materia medica from the perspective of the “property-effect-component” theory. The research and development of new TCM drugs should be based on the TCM theory in disease explanation and highlight the characteristics of TCM with the path of “disease-syndrome-prescription-medicine-technology-agentia.” That is, the development of new drugs should follow the basic framework and theoretical system of TCM, choose the right research path, and determine reasonable extraction, purification, and preparation technology [25].

### 2.3. Efficacy Evaluation-Outcome of Disease or Improvement of Syndrome?

An NIH report pointed out that efficacy evaluation is a key and core issue for traditional remedies [26]. The evaluation of the TCM clinical curative effect measures the efficacy of prescription, acupuncture, and moxibustion on the human body through TCM observation, listening, inquiring, feeling the pulse, principles of formulating prescriptions, and treatments based on syndrome differentiation. According to Western medical standards, the evaluation of the clinical efficacy of drugs is the outcome of the disease, but CHM has its particularity. One Chinese herbal formula can be used for several Western diseases [27]. Taking Liuwei Dihuang Pills as an example, its efficacy is not specific to one kind of disease but to a series of syndromes, such as dizziness and tinnitus, soreness, and weakness of the waist and knees, hectic fever, night sweat, spermatism, drinking, and urine, which are caused by a deficiency of kidney yin. Pharmacological studies have demonstrated the efficacy of this formulation for a variety of diseases, including metabolic syndrome, diabetes, and cardiovascular disease [28]. TCM clinical practice is based on the theoretical system of “syndrome differentiation,” and its efficacy evaluation should not be separated from the improvement of the syndrome. In the fourth chapter of guiding principles for “Clinical Research of New Chinese Materia Medica (Trial)” issued by China in 2002, the methods for efficacy evaluation of the syndromes and symptoms in clinical trials of CHM were discussed [29]. Zhang et al. [30] proposed that the evaluation of the clinical efficacy of TCM should be classified according to the main direction of efficacy. CHM can be roughly divided into three categories: first, to treat diseases, such as coronary heart disease, angina pectoris, urinary tract infection, and blood lipid; second, to improve some symptoms, such as headache, insomnia, and cough; and third, to improve certain syndromes, such as liver-stomach disharmony syndrome and excessive heat-toxin syndrome. For the first kind of drug, the efficacy evaluation should mainly refer to Western disease diagnosis and treatment standards, with the supplement of syndrome improvement. For the second kind, symptom improvement should be emphasized. For the third kind, they should be evaluated mainly based on syndrome improvements, and the evaluation of the disease outcome should be weighted to a lesser extent.

At present, TCM clinical efficacy evaluations include quantitative and qualitative analyses with the following aspects: symptom and sign improvement, physical and chemical indicator improvement, syndrome improvement, the incidence of important clinical events, patient report outcomes, caregiver reports, safety evaluations, and health economic evaluations. Biomedical indicators are measurable. Symptoms and signs, such as the degree of pain, can be quantified by an international unified scale. For functional diseases, such as chronic fatigue syndrome and menopausal syndrome, laboratory indicators cannot reflect the positive outcome, but the patient's self-reported symptoms and psychological state can provide reliable evidence, which makes patient-reported outcomes (PROs) a unique indicator of patients' evaluation of the intervention. In addition, some subjective feelings can be qualitatively described in face-to-face interviews before and after the intervention. Qualitative indicators can supplement quantitative analysis and more comprehensively reflect the efficacy and characteristics of TCM clinical research. Liu et al. [31] drew lessons from the thought of evaluating athletes' skills in sports competitions and proposed a new strategy from evaluating “doctor” to evaluating “prescription,” which combined clinical epidemiology and evidence-based medicine with syndrome differentiation. The evaluation process should also adhere to the basic principles of control, adopt the appropriate design methods, such as RCT and cohort study, and select the suitable method according to the research purpose combining the “full sample, mixed big data” and “sampling, precise small data” in RWS. The evaluation results first recommend the appropriate “doctor”, rather than a certain “prescription” to treat a certain disease. The comprehensive curative effect of the “representative team” is used to reflect the clinical effectiveness of syndrome differentiation in treating certain diseases. The ideas and strategies of this study break through the conventional clinical evaluation method and open a new way for the clinical efficacy evaluation of TCM individualized diagnosis and treatment.

## 3. Risk Assessment

Risk assessment is one of the most important considerations in the ethical review of human research. The principles focus on risk minimization and a reasonable balance between potential risks and benefits. In contrast to chemical drugs, CHM has a long history in clinical practice, even for hundreds or thousands of years. It seems reasonable to assume that information about risks and potential side effects has been gathered according to years of experience. Such information evolves from generation to generation and is crucial to forming local traditional medicine. There is a record in ancient Chinese books that Shen Nong suffered 70 poisons in trying 100 herbs in one day, which allowed him to discover the efficacy of CHM and was the start of a toxicological study on TCM. The related records of toxic traditional Chinese medicine were found in the books of Shen Nong's Herbal Classic, Compendium of Materia Medica, and Chinese materia medica. The law of Chinese Pharmacopoeia (2015 edition) includes 83 kinds of toxic Chinese medicinal materials and prepared herbal medicine in slices, with information about their toxicity and attention in clinical use. Ten of them are labeled “extremely toxic,” 42 are “toxic,” 31 are “mildly toxic.” The law also includes 99 kinds of herbs and prepared herbal medicine in slices that are forbidden or used with caution in pregnant women [32]. The understanding of the toxicity of CHM originated from clinical experience by generations, whereas these data can be used as preclinical references. According to international principles, a large proportion of TCM studies can start directly at the clinical trial stage by providing information on toxicology and safety dosage, and the requirement of laboratory research may not be necessary or be simplified at the beginning of clinical drug development processes [33]. Risk assessment may be acceptable when herbs are tested in the same formulation at the dose and duration no longer than their historical and traditional usage [34]. However, the results cannot be predicted when the formula is applied to a completely different environment or conditions without traditional roots [35]. Moreover, even well-studied herbs may neglect vulnerable groups of people, such as children, pregnant women, or elderly people with basic diseases [36]. Their potential risks are often unknown and have a direct impact on the informed consent process [37].

In China, cultural factors may influence judgments about the risks and benefits of herbal research. People may accept the safe use assumptions of CHM based on cultural familiarity and historical use. It is commonly believed that natural herbs that have been tested for thousands of years are safer than synthetic drugs. In fact, anything that affects the body's defense or physiology may have varying degrees of side effects. At the same time, the extensive use of herbal medicines in China may cause herbal consumers and even healthcare providers to ignore the potential side effects, leading to underreporting of adverse drug reactions in local herbal use [38]. With the wide application of CHM in clinical practice, reports of adverse reactions are common. These adverse reactions, especially serious adverse reactions, arouse people's concern and worry about the safety of CHM. Clinical adverse reactions to CHM involve tissues and organs in the whole body, and serious adverse reactions may even endanger life. For example, herbal medicine with aconitum alkaloids, which are mainly composed of diester alkaloids, can cause cardiovascular adverse reactions, such as arrhythmias, palpitations, and chest distress [39]. Long-term use of herbs containing aristolochic acid may cause acute or subacute progressive and irreversible renal injury [40]. The oral administration of *Polygonum multiflorum* may have the risk of liver injury, which mainly causes digestive tract symptoms, such as general fatigue, loss of appetite and oiliness, jaundice with yellow urine, eyes, and skin, and abnormal laboratory examination, such as elevated bilirubin and transaminase [41].

According to “Ethical Guidelines for Clinical Research Involving Humans (2020)” [42] in China, compound Chinese medicine preparation which comes from ancient classic prescriptions or indications for improving the syndrome with clinical application basis does not require animal efficacy tests. But nonclinical safety studies must be conducted to ensure drug safety. If TCM decoction contains toxic Chinese medicine or the dosage exceeds the Chinese Pharmacopoeia, verifiable safety data in vitro or animal experiments or relevant preclinical study results should be provided. Certain studies may be necessary to complement the human experience, especially to detect chronic toxicity that may occur after the long-term use of herbs. If the method of preparation, route of administration, dosage form, or duration of herbs changed, additional basic medical studies, such as toxicological studies, may be necessary prior to clinical trials in addition to empirical evidence [43, 44]. In general, current clinical trial protocols largely address the problem of monitoring indexes as well as emergency treatment plans for early warning of known toxicity. However, early warning of unknown toxicity is an advanced awareness to protect participants' rights, which happens to be an important scientific and ethical issue to ensure medication safety and protect participants' rights and interests. We believe that it is necessary to draw the attention of researchers and sponsors to whether the tested formulation contains toxic drugs, eighteen incompatible medicaments, nineteen mutual restraint medicaments, contraindications during pregnancy, drugs with a strong effect of invigorating blood circulation and eliminating stasis, or drugs belonging to the same species of toxic drugs. In the ethical review, the researchers, according to these, should audit, in order to avoid harm to the subjects. Attention to potential toxicity is also warranted to protect the interests of the participants to the greatest extent.

The potential risks and adverse reactions should be clearly stated in the informed consent form. In addition to being informed of the risks associated with the study, participants should also be monitored for adverse medical events throughout the study. According to the theory of TCM, the syndromes of diseases are constantly changing in the process, regardless of whether there is any noncorrespondence between “medicine” and “syndrome” when treating with a fixed prescription for a longer period of time, which is quite common in clinical practices. An evaluation should be conducted after each therapeutic intervention and before the next phase of intervention to investigate the presence of adverse reactions or other discomforts. All suspected adverse events should be reported promptly and evaluated accordingly. There are also neglected risks of making “effective” judgments as an “expert.” Ethics committee members with a TCM background tend to agree with the positive effect of TCM in treating advanced malignant tumors, such as reducing the toxicity and side effects of radiotherapy and chemotherapy drugs, prolonging the survival period of patients, and improving their quality of life. Therefore, it is easy to conclude that the project has “scientific design, sufficient assumptions, reasonable risks, and benefits,” which is acceptable in terms of scientific and ethical rationality. However, in view of a TCM “layperson” or a Western medicine doctor who has never learned TCM, long-term use of CHM with unclear ingredients on patients with advanced cancer may cause great risks. This reminds the ethics committee to use objective thought instead of inherent ideas to avoid exaggerating risks from laypersons and neglecting risks from experts. It is extremely necessary to equip people with different backgrounds on an ethical review team and engage in active discussions in the ethical review.

## 4. Informed Consent

Valid informed consent is important to protect participants' rights and interests. Early in 1947, the Nuremberg Code stated that “the voluntary consent of the human subject is absolutely essential.” Informed consent has become a basic ethical requirement for biomedical research involving human beings. Informed consent includes two parts: informed and consent. Informed means that sufficient information and the ability to understand are required to make rational decisions. Consent refers to the free will of the participants and the ability to make a decision about whether to participate in the research. Informed consent is necessary, but it does not mean that research with a consent form is valid. The current consensus on informed consent in biomedical research is that we not only need formal informed consent (a signed informed consent form) but also must meet substantive ethical standards. To meet the standard of substantive ethics, the following three conditions must be met: (1) provide participants with adequate, accurate, and complete information, with no deception, hiding, or distortion; (2) help participants understand the information being provided; (3) ensure that the consent of the participants is of free will without coercion or improper inducement. Whereas many researchers do obtain written consent from participants, the validity of substantive ethics is questionable from practical experience. This is a common problem associated with informed consent in biomedical research involving people, and TCM clinical research is no exception. The statement “there is no risk in this research” is often used by researchers to justify the exemption from informed consent, especially when analyzing retrospective medical records or using residual biological samples. Some researchers or participants confuse informed consent with research and clinical treatment. Some participants sign the informed consent form because of their trust in the researcher's doctor identity. These are issues that are easily overlooked in formal ethical reviews.

The subject of informed consent is vague in China. In China, both Western medicine and Traditional Chinese medicine tend to solicit the opinions of the subjects' family members in studies with certain risks. In the Western value system, a person is an independent individual with the ability to make decisions, and the object of informed consent in clinical research is the participants themselves. In China, however, based on the influence of the home-oriented view from traditional Confucianism, it varies with objective reality to be an isolated atomic individual. A relationship is a social attribute of a person, and individual decisions are influenced by the family. In particular, when the subject is in a state of illness, ignoring the opinions of family members can arouse controversy. For example, if the subject agrees to participate in the study but the family members disagree, should the opinions of the family members be adopted? Or is it allowable for family members to agree to participate but in some altruistic way to hide the truth from the subject in the research of some new drug or new treatment? Generally, it seems more consistent with traditional Chinese values to seek the mutual consent of the subjects and their families from the perspective of relational autonomy. Regardless of the purpose, it is ethically untenable to deceive people into signing a so-called informed consent form. Special attention should be given to ethical reviews of clinical trials.

The complexity and abstractness of TCM theories and the inherent impression of “relatively small side effects of TCM” in people's habitual thinking mode weaken the cognitive ability of participants to some extent. Therefore, in the process of obtaining the informed consent of the subjects, the researchers should explain to the participants item by item according to the contents of the informed consent and use the language and words that are easy to understand, especially in the explanation of the TCM specific terms and theories. Due to the great development of modern research on TCM and the internationalization of TCM culture, the risks associated with TCM clinical researches are understandable after certain procedures are explained properly.

## 5. Placebo Use

The use of placebo in RCTs is also a major concern of ethical reviews in TCM clinical trials. It is not ethical to treat patients with a placebo alone when it is clear that an effective treatment is available to prevent disease progression. At present, the following recommendations are generally followed on whether placebos can be used in TCM clinical studies: (1) the disease under research is self-limited and can be cured even without treatment, (2) there is no specific treatment for the research disease at present, which means new treatment should be explored, and (3) chronic disease and short-term absence of treatment will not significantly affect the prognosis.

In 2004, the American Food and Drug Administration stated in a new drug study that randomized, double-blind, placebo studies have the highest level of clinical evidence, and placebos should be used in botanical studies unless there are sufficient reasons not to do so [45]. Therefore, using a placebo in TCM for the observation of clinical efficacy and adverse reactions, especially in the research and development of new drugs, is not only a standard requirement of clinical randomized control but also one of the effective ways for TCM to prove its scientific value to the world. Placebo use is one of the keys to the successful implementation of the blinding method. However, blindness is easily broken in the process of the trial without careful design. A placebo should be completely the same as the experimental drug in terms of packaging, appearance, odor, and even taste so that experimenters and participants can hardly distinguish the test drug and a placebo from vision or taste. The traditional dosage form of TCM is decoction. Nevertheless, decoction brings great difficulties to the preparation of a placebo due to its special color, smell, and taste. In the meantime, although capsule preparation can reduce the interference of appearance, smell, and taste, capsules have their own limits in Chinese herbal research because of the difficulty of extracting all effective substances. At present, a placebo of TCM decoction is usually prepared by adding excipients, essence, and pigment. Fu [46] collected 231 randomized controlled studies on TCM placebo from 1979 to 2008; 60.17% of the studies described the preparation of placebo, but there was no confirmation that the preparations were totally similar to placebo in all aspects.

To maximize benefits and minimize risks to participants, the following aspects should be considered in the ethical review of TCM clinical trials: (1) calculate and minimize sample content, so as to avoid too many participants receiving treatment with poor efficacy or adverse reactions, and ensure the rights and interests of participants; (2) ensure the safety and reliability of auxiliary materials or raw materials of placebos; in the manufacturing process of TCM placebo, the relevant materials should be in line with the relevant international standards to guarantee the quality and safety of placebos; (3) pay close attention to the occurrence of adverse events, including serious ones; establish the standard operating procedure of the corresponding first aid measures and reasonable termination criteria; (4) an add-on design scheme can be adopted, where the experimental group and control group can adopt standard therapy as a basic intervention and use TCM decoction and placebo, respectively, to minimize harm.

Another ethical problem that is often ignored is participant preferences in RCTs. TCM is generally considered an effective treatment, and participants tend to accept their preferred treatments, rather than being randomly assigned [47, 48]. Some RCTs have been modified to allow for participant preference issues, including the Baskerville design and comprehensive cohort design [49]. The Baskerville design allows participants to choose whether they agree to stay in a randomly assigned group, switch to another group, or drop out of the trial. A comprehensive cohort design allows participants to freely choose whether they want to enter the randomization process before they are randomly assigned [24]. Thus, it will be difficult to enroll a sufficient number of participants in the control group because most participants tend to choose to enroll in the treatment group. Further studies are needed before patient preference trials are widely undertaken.

We agree with some experts that in the ethical review of TCM clinical trials, in addition to the modern scientific view, we should consider more of the cultural connotation and theory of TCM. Modern technology and methods should not replace the culture of TCM. At present, almost all TCM clinical trials are written in accordance with Good Clinical Practice (GCP), which lacks the characteristics of TCM. More attention should be given to the realization of guidance from Chinese culture to TCM research methods. The ethics committee should broaden the staff composition with more people with a TCM background or conduct TCM-related training. More international communications are also necessary to supplement cross-cultural experiences.

## Data Availability

No data were used to support this study.
